# Hypertension and Its Associated Risk Factors in the Kingdom of Saudi Arabia, 2013: A National Survey

**DOI:** 10.1155/2014/564679

**Published:** 2014-08-06

**Authors:** Charbel El Bcheraoui, Ziad A. Memish, Marwa Tuffaha, Farah Daoud, Margaret Robinson, Sara Jaber, Sarah Mikhitarian, Mohammad Al Saeedi, Mohammad A. AlMazroa, Ali H. Mokdad, Abdullah A. Al Rabeeah

**Affiliations:** ^1^Institute for Health Metrics and Evaluation, University of Washington, 2301 Fifth Avenue, Suite 600, Seattle, WA 98121, USA; ^2^Ministry of Health of the Kingdom of Saudi Arabia, Assadah, Al Murabba, Riyadh 12613, Saudi Arabia

## Abstract

Current data on hypertension in the Kingdom of Saudi Arabia are lacking. We conducted a national survey to inform decision-makers on the current magnitude of the epidemic. We measured systolic and diastolic blood pressure of 10,735 Saudis aged 15 years or older and interviewed them through a national multistage survey. We used multivariate logistic regressions to describe sociodemographic characteristics and risk factors of hypertensive, borderline hypertensive, and undiagnosed hypertensive Saudis. We found that 15.2% and 40.6% of Saudis were hypertensive or borderline hypertensive, respectively. Risk of hypertension increased among men, with age, obesity, diabetes, and hypercholesterolemia. 57.8% of hypertensive Saudis were undiagnosed. These were more likely to be male, older, and diagnosed with diabetes. Among participants diagnosed with hypertension, 78.9% reported taking medication for their condition. About 45% of participants on medication for hypertension had their blood pressure controlled. The prevalence of hypertension and borderline hypertension is very high in Saudi Arabia. Moreover, control of hypertension is poor. With the majority of hypertensive Saudis being unaware of their condition, a national plan is needed to increase utilization of freely available screening, preventive, and medical services.

## 1. Introduction

Hypertension is a leading risk factor for morbidity and mortality [1]. Untreated hypertension may lead to many serious health conditions, including stroke, aneurysms, hypertensive heart disease, coronary artery disease, kidney disease, or peripheral artery disease [2–4]. Hypertension has a major economic impact ranging from medical costs to human capital loss and decrease in productivity [5, 6].

The Global Burden of Disease 2010 (GBD 2010) study estimated that hypertension was the leading risk factor for death in the Kingdom of Saudi Arabia (KSA) [7]. Hypertension accounted for about 24% of total deaths from cardiovascular and circulatory diseases and 1.87% of total deaths from hypertensive urogenital, blood, and endocrine diseases [8]. From 1990 to 2010 the burden of hypertension remained very high in KSA [7]. Previous studies reported high levels of blood pressure in KSA. These levels ranged from 26.1% among individuals 30–70 years old in 1995–2000 [9] to 25.5% among individuals 15–64 years old in 2005 [10, 11].

In order to assess the current status of hypertension in KSA, we conducted a large national survey. We selected a national sample to be representative of each of the 20 KSA health regions and the kingdom. We used an adapted standard questionnaire and took physical measurements and blood samples to examine blood pressure and chronic diseases. We used computer assisted personal interviewing to conduct the survey. The software used for offline data collection allowed interviewers to upload data to our servers on daily basis and hence a rigid monitoring of data quality.

## 2. Materials and Methods

The Saudi Health Information Survey (SHIS) is a national multistage survey of individuals aged 15 years or older. Households were randomly selected from a national sampling frame maintained and updated by the Census Bureau. KSA was divided into 13 regions. Each region was divided into subregions and blocks. All regions were included, and a probability proportional to size was used to randomly select subregions and blocks. Households were randomly selected from each block. A roster of household members was conducted and an adult aged 15 or older was randomly selected to be surveyed. Weight, height, and blood pressure were measured at the household by a trained professional. Omron HN286 (SN: 201207-03163F) and Omron M6 Comfort (HEM-7223-E) were used to measure weight and blood pressure.

The survey included questions on sociodemographic characteristics, tobacco consumption, diet, physical activity, health care utilization, different health-related behaviors, and self-reported chronic conditions.

We used measured weight and height to calculate body mass index (BMI) as weight (kg)/height (m^2^). Participants were classified into four groups: (1) underweight: BMI < 18.5; (2) normal weight: BMI within 18.5–25.0; (3) overweight: BMI within 25.0–30.0; or (4) obese: BMI was greater than or equal to 30.0. Respondents were considered to be current smokers if they reported ever smoking any tobacco products and still currently smoke tobacco and past smokers if they reported smoking in the past but not anymore. We computed the servings of fruits and vegetables and red meats and chicken consumed per day from the detailed dietary questionnaire as the sum of the average daily consumption of fruits, fruit juices, and vegetables and red meats and chicken. We used the International Physical Activity questionnaire [12] to classify respondents into four groups of physical activity: (1) met vigorous physical activity, (2) met moderate physical activity, (3) insufficient physical activity to meet vigorous or moderate levels, and (4) no physical activity.

To assess diagnosed hypertension, diabetes, and hypercholesterolemia status, respondents were asked three separate questions: “Have you ever been told by a doctor, nurse, or other health professional that you had: (1) high blood pressure, otherwise known as hypertension; (2) diabetes mellitus, otherwise known as diabetes, sugar diabetes, high blood glucose, or high blood sugar; (3) hypercholesterolemia, otherwise known as high or abnormal blood cholesterol?” Women diagnosed with diabetes or hypertension during pregnancy were counted as not having these conditions. Those who were diagnosed with either of these conditions were further asked if they are currently receiving any treatment for their condition. Similarly, the same type of questions was used to determine previous diagnosis of stroke, myocardial infarction, atrial fibrillation, cardiac arrest, congestive heart failure, chronic obstructive pulmonary disease, asthma, renal failure, and cancer. We considered a person to be diagnosed with a chronic condition if they reported being diagnosed with any of the conditions cited earlier.

A total of three blood measurements were taken with the participant resting and at five-minute intervals. We followed the National Health and Nutrition Examination Survey (NHANES) for determining blood pressure levels [13]. Basically, respondents were considered to have hypertension if they met any of the following criteria: (1) measured diastolic or systolic blood pressure exceeding 89 or 139 mmHg, respectively, or (2) measured diastolic or systolic blood pressure not exceeding the appropriate threshold, but the respondent reported taking medications for hypertension. Hence, respondents who were on drugs for hypertension were considered hypertensive even if their measured diastolic or systolic blood pressure did not exceed 89 or 139 mmHg, respectively. Respondents were considered to have borderline hypertension if (1) they did not report taking drugs for hypertension and (2) their measured diastolic blood pressure was between 80 and less than 90 mmHg or systolic blood pressure was between 120 and 139 mmHg.

We used SAS 9.2 (SAS Institute Inc., Cary, NC, USA) for analyses and to account for the complex sampling design.

## 3. Results

Between April and June 2013, a total of 12,000 households were contacted and a total of 10,735 participants completed the survey (response rate of 89.4%). The characteristics of respondents who completed the questionnaire are presented in Table 1.

Overall, 917,188 (7.1%) Saudis reported a diagnosis of hypertension. A total of 1,957,191 (15.2%) Saudis aged 15 years or older had hypertension (measured or reported taking blood pressure medication). Of these, 1,119,027 were undiagnosed. Moreover, 40.6% of Saudis, or 5,222,051, had borderline hypertension. Characteristics of respondents with undiagnosed hypertension, hypertension, and borderline hypertension are presented in Table 2.

**Figure 1:**
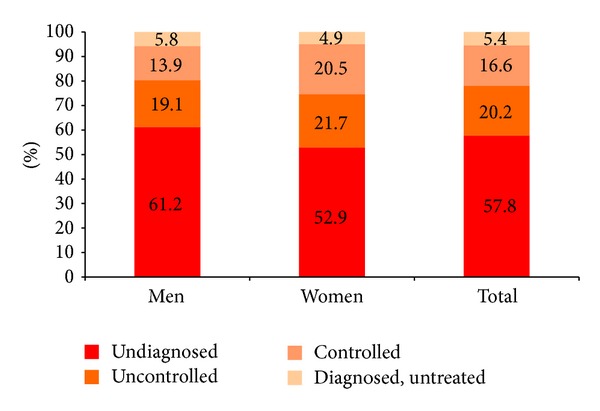
Percent distribution of diagnosis and treatment status among hypertensive Saudis aged 15 years or older, 2013.

Among participants diagnosed with hypertension, 78.9% reported taking medication for their condition. About 45% of participants on medication for hypertension had their blood pressure controlled. Hence, about 390,338 adults had uncontrolled blood pressure. Among all those who are hypertensive, 57.8%, 20.2%, 16.6%, and 5.4% are undiagnosed, treated uncontrolled, treated controlled, and untreated, respectively (Figure 1).

Age, sex, and diagnosis history of diabetes and hypercholesterolemia were associated with hypertension (Table 3). The risk of being hypertensive was lower among females (AOR = 0.61; 95% CI: 0.50–0.74) but increased with age (AOR = 1.07; 95% CI: 1.06–1.08), among obese participants (AOR = 2.24; 95% CI: 1.89–2.65) and those who have been previously diagnosed with diabetes (AOR = 1.95; 95% CI: 1.57–2.43) and hypercholesterolemia (AOR = 1.94; 95% CI: 1.51–2.47). On the other hand, marital status, education, smoking status, diet, time spent watching TV, levels of physical activity, diagnosis history of prediabetes, or other chronic conditions were not associated with the risk of hypertension (Table 3). Being male, older, and obese and having a diagnostic history of diabetes also increased the risk of borderline hypertension. Daily consumption of two to three servings of red meats and chicken and being moderately active was associated with the risk of borderline hypertension (Table 4).

A large percentage of hypertension was undiagnosed, as 57.8% of those with hypertension did not know of their condition, a total of 1,119,027 Saudis. The likelihood of being undiagnosed decreased among women (AOR = 0.54; 95% CI: 0.44–0.67) and increased with age (AOR = 1.05; 95% CI: 1.05-1.06) and diagnosis history of diabetes (AOR = 1.46; 95% CI: 1.09–1.94) (Table 5).

## 4. Discussion

Our study revealed high rates of hypertension and borderline hypertension in KSA. Moreover, our findings revealed high rates of uncontrolled hypertension in KSA. Our findings are striking in a country with free medical care and high resources. Indeed, these findings call for action to control the burden of hypertension in the kingdom. A national plan to increase awareness, early detection, and control of hypertension is urgently needed.

Very few studies previously reported on hypertension in KSA. The most recent estimates date back to 2005 and provided a prevalence of 11.5% of reportedly diagnosed hypertension, among individuals aged 15–64 years [10], a much higher prevalence than 5.6% from our study. Data from 1995–2000 for Saudis aged 30 years or older showed a hypertension prevalence of 26.1% [9]. In comparison, we found that 27.2% of those aged 30 or older had hypertension. There are several factors that could explain these differences. First, our study is national and applied standardized methodology for data collection. Second, we used weighted analyses to generalize our findings. However, the 2005 STEPS survey and our study should be comparable and possibly indicate a leveling of the hypertension prevalence in KSA.

Our results for increased risk of hypertension with age and among men are similar to previous studies from KSA and other countries. However, literature on marital status and hypertension is inconclusive and mostly focused on comparing currently married to never married persons [14, 15]. Our study did not show any association between marital status and high blood pressure after adjusting for confounders. This is contrary to what [9] had been reported previously on this association.

Interestingly, hypertension among Saudis did not vary with educational levels. Previous reports on association between education and hypertension are mixed as some studies have reported the lack of association [16] or an inverse relationship [17, 18].

While the findings on hypertension might be pointing toward stabilization or decline in prevalence, the KSA health system still has many challenges. For instance, the majority (57.8%) of hypertensive Saudis are undiagnosed. The other 55.0% of those on treatment were not controlled. SHIS included questions on health care utilization, and only 14.8% reported visiting a health clinic for a regular checkup within the last year. It is probable that Saudis are not engaged in preventive health care and only seek medical care for illnesses. Understanding the barriers to seeking care is crucial in order to improve the health of Saudis.

Our study has some limitations. First, our data are cross-sectional, and hence we cannot assess causality. Second, many of our behavioral data, such as diet and physical activity, are self-reported and subject to recall and social desirability biases. On the other hand, our study is based on a large sample size and used a standardized methodology for all its measures.

Despite these limitations, our study remains nationally representative and has the merit of providing accurate data due to our near-real-time data quality monitoring through the whole survey period. The physical and blood measurements allowed us to control recall bias regarding diagnosed diseases and to uncover respondents who were affected by these chronic diseases but undiagnosed.

## 5. Conclusions

Our findings from this study along with those we reported previously in GBD 2010 [7] call for a national plan to prevent and control the burden of hypertension in KSA. Indeed, the plan has to be comprehensive to include programs to improve health behaviors such as diet and physical activity. Moreover, the plan should increase health care utilization for preventive services. As uncontrolled blood pressure leads to catastrophic events such as stroke, heart attack, and renal failure [2], physicians in KSA should be encouraged to monitor their patients to ensure that their blood pressure is controlled. Saudis should be encouraged to monitor their own blood pressure and seek medical care to control their conditions.

KSA has a young population with 81% of the population under the age of 40 [19]. GBD 2010 reported that life expectancy has increased from 72.5 and 76.3 years in 1990 to 75.0 and 79.9 years in 2010 for men and women, respectively [20]. As life expectancy increases and the size of population is on the rise, the burden of hypertension and other chronic diseases, if uncontrolled, will pose major challenges to the health system. Prevention should be a priority for all in KSA. As the KSA MOH has successfully tackled the burden of infectious diseases through prevention, the same effort should be applied to reduce the burden of chronic diseases.

## Figures and Tables

**Table 1:** Sociodemographic characteristics, in Saudi Arabia, of males and females age 15 years or older, 2013.

Sociodemographic and risk factors	Categories	Males			Females		
		*N*	Weighted %	SE	*N*	Weighted %	SE
Age (years)	15–24	1189	40.79	1.03	1193	39.81	1.03
	25–34	1254	21.71	0.75	1503	21.28	0.71
	35–44	1132	13.59	0.53	1207	16.80	0.62
	45–54	722	11.97	0.55	798	12.80	0.57
	55–64	439	7.07	0.44	423	5.85	0.39
	65+	517	4.87	0.29	358	3.46	0.26

Marital status	Currently married	3514	49.31	0.98	3462	49.40	0.97
	Never married	1569	49.26	0.99	1260	42.38	1.03
	Separated, divorced, or widowed	159	1.43	0.15	738	8.22	0.43

Education	Primary school or less	1217	20.26	0.75	2069	32.52	0.88
	Elementary or high school completed	2745	59.34	0.94	2127	46.17	0.98
	College degree or higher education	1282	20.40	0.72	1275	21.31	0.77

SE: standard error.

**Table 2:** Sociodemographic characteristics of undiagnosed hypertensive, hypertensive, and borderline hypertensive Saudis, males and females aged 15 years or older, 2013.

Sociodemographic and risk factors	Undiagnosed hypertensive						Hypertensive						Borderline hypertensive					
	Males			Females			Males			Females			Males			Females		
	*N*	Weighted %	SE	*N*	Weighted %	SE	*N*	Weighted %	SE	*N*	Weighted %	SE	*N*	Weighted %	SE	*N*	Weighted %	SE
Age (years)																		
15–24	48	4.22	0.76	22	2.38	0.63	50	4.27	0.75	24	2.47	0.63	454	40.44	1.87	295	25.13	1.65
25–34	104	8.01	0.95	57	3.46	0.62	135	10.36	1.07	69	4.16	0.66	651	60.77	1.96	485	35.87	1.79
35–44	160	15.39	1.42	97	8.45	1.12	216	21.13	1.61	149	13.35	1.38	612	67.45	2.06	501	49.05	2.04
45–54	136	21.73	2.12	103	12.20	1.43	232	35.36	2.37	224	26.93	2.04	373	72.81	2.54	366	59.61	2.60
55–64	93	20.14	2.57	84	21.50	2.98	209	48.36	3.17	214	48.61	3.45	224	86.63	2.33	186	67.36	4.09
65+	117	26.44	2.72	68	19.65	3.14	318	68.09	2.66	217	60.99	3.62	201	75.00	4.14	141	68.59	4.85

Marital status																		
Currently married	539	16.51	0.85	276	8.52	0.66	994	29.33	1.03	528	16.16	0.87	1790	68.60	1.19	1324	46.62	1.21
Never married	85	4.88	0.70	40	2.82	0.62	107	5.90	0.74	52	3.21	0.64	646	44.11	1.69	329	26.13	1.61
Separated, divorced, or widowed	34	23.87	4.58	113	14.88	1.85	58	35.38	4.91	314	39.55	2.58	73	74.45	5.18	313	60.84	3.22

Education																		
Primary school or less	209	15.98	1.42	269	11.91	1.02	449	30.90	1.78	634	25.69	1.33	554	56.15	2.53	842	52.14	1.81
Elementary or high school completed	317	9.26	0.71	93	4.12	0.57	473	13.34	0.81	166	6.47	0.67	1267	52.37	1.46	687	30.19	1.41
College degree or higher education	132	10.59	1.13	69	3.90	0.62	236	18.00	1.37	97	5.65	0.74	692	62.89	2.12	442	37.19	2.03

Smoking status																		
Never smoked							761	16.06	0.76	870	12.37	0.57	1703	53.41	1.33	1913	37.91	1.01
Exsmoker							135	31.87	3.11	5	5.33	3.01	182	56.15	3.86	15	44.06	13.70
Current smoker							259	18.78	1.47	22	27.41	7.29	624	60.36	2.23	41	48.13	8.43

Daily servings of fruits and vegetables																		
0							33	19.98	4.62	32	11.18	2.56	52	43.47	6.30	45	31.58	5.31
0–3							846	17.19	0.76	711	12.33	0.64	1950	55.09	1.27	1553	37.97	1.12
3–5							165	19.08	1.88	87	13.68	1.89	298	56.43	3.03	214	43.36	3.15
5+							96	21.56	2.65	47	13.04	2.48	176	61.07	3.91	115	34.76	3.85

Daily servings of red meat and chicken																		
0-1							328	20.84	1.41	345	13.21	0.95	652	60.56	2.14	734	38.41	1.62
1-2							405	16.93	1.07	317	12.02	0.96	977	54.37	1.78	687	37.94	1.68
2-3							206	16.52	1.56	111	12.55	1.72	424	46.41	2.63	267	35.70	2.78
3+							207	16.23	1.53	106	11.62	1.48	440	55.58	2.60	255	39.20	2.74

Daily hours spent watching TV																		
0-1							96	30.51	3.80	103	27.14	3.26	127	60.20	5.26	147	47.61	4.20
1–3							402	20.00	1.24	289	13.39	1.04	846	57.92	1.92	609	35.72	1.69
3–5							244	15.49	1.31	167	10.24	1.11	608	56.20	2.25	450	39.45	2.19
5+							163	14.74	1.49	150	12.22	1.47	380	54.47	2.74	298	37.42	2.57

Levels of physical activity																		
None							366	20.95	1.39	522	14.11	0.86	729	62.20	2.08	1066	40.86	1.43
Low							287	22.15	1.57	212	12.65	1.11	618	59.41	2.31	476	37.43	1.98
Moderate							188	18.30	1.74	66	9.04	1.46	383	53.81	2.72	168	30.74	2.88
High							319	13.10	0.98	97	9.75	1.46	785	49.28	1.85	264	36.16	2.53

Obesity																		
Not obese							628	12.85	0.67	327	7.14	0.56	1749	50.95	1.27	1055	32.36	1.21
Obese							486	32.17	1.72	529	22.08	1.23	719	71.93	2.04	867	51.97	1.72

History of diagnosis with prediabetes																		
No	633	10.93	0.57	408	6.51	0.46	1075	17.34	0.68	808	11.90	0.58	2403	54.92	1.12	1901	37.96	1.01
Yes	13	11.44	4.00	14	11.00	3.22	46	43.48	6.39	47	41.02	6.51	49	68.35	8.11	35	48.94	8.44

History of diagnosis with diabetes																		
No	527	10.06	0.58	346	6.07	0.46	791	14.30	0.66	574	9.18	0.53	2184	53.69	1.16	1724	36.48	1.03
Yes	130	19.26	2.00	82	13.82	1.96	362	51.72	2.58	314	56.60	2.88	310	75.11	3.05	226	67.31	3.48

History of diagnosis with hypercholesterolemia																		
No	582	10.38	0.56	383	6.38	0.46	872	14.79	0.65	661	10.19	0.55	2277	54.15	1.15	1812	37.14	1.02
Yes	60	14.64	2.42	37	9.23	2.06	239	54.46	3.19	181	55.04	3.77	193	73.31	3.62	119	61.42	4.26

Diagnosis of chronic condition																		
No	620	10.96	0.58	403	6.43	0.45	1010	16.62	0.68	791	11.55	0.56	2354	54.94	1.14	1871	37.93	1.02
Yes	38	9.39	2.15	28	9.83	2.98	150	34.09	3.30	103	30.68	3.96	159	57.08	4.20	99	41.20	4.58

SE: standard error.

**Table 3:** Multivariate logistic regression for sociodemographic characteristics, risk factors, and hypertension, in Saudi Arabia, of males and females aged 15 years or older, 2013.

Socio-demographic and risk factors	Categories	Sociodemographic model		Full adjusted model	
		AOR	95% CI	AOR	95% CI
Sex	Males	REF		REF	
	Females	**0.61**	**0.52–0.72**	**0.61**	**0.50–0.74**

Age∗		**1.08**	**1.07–1.09**	**1.07**	**1.06–1.08**

Marital status	Currently married	REF		REF	
	Never married	0.86	0.66–1.12	1.05	0.79–1.39
	Separated, divorced, or widowed	1.29	1.00–1.66	1.28	0.98–1.68

Education	Primary school or less	REF			
	Elementary or high school completed	1.01	0.82–1.24		
	College degree or higher education	0.86	0.69–1.07		

Smoking status	Never smoked			REF	
	Exsmoker			1.26	0.83–1.93
	Current smoker			1.21	0.95–1.55

Levels of physical activity	None			REF	
	Low			1.19	0.97–1.46
	Moderate			1.09	0.83–1.42
	High			1.07	0.84–1.35

Obesity	Not obese			REF	
	Obese			**2.24**	**1.89–2.65**

History of diagnosis with diabetes	No			REF	
	Yes			**1.95**	**1.57–2.43**

History of diagnosis with hypercholesterolemia	No			REF	
	Yes			**1.94**	**1.51–2.47**

Diagnosis of chronic condition	No			REF	
	Yes			1.28	0.93–1.76

*AOR for age should be considered as for an increase of one year.

Odds ratios were adjusted for sociodemographic characteristics: sex, age, marital status, and education.

AOR: adjusted odds ratios; CI: confidence intervals; REF: reference.

**Table 4:** Multivariate logistic regression for sociodemographic characteristics, risk factors, and borderline hypertension, in Saudi Arabia, of males and females aged 15 years or older, 2013.

Sociodemographic and risk factors	Categories	Sociodemographic model		Full adjusted model	
		AOR	95% CI	AOR	95% CI
Sex	Males	REF		REF	
	Females	**0.44**	**0.38–0.50**	**0.36**	**0.31–0.43**

Age∗		**1.04**	**1.03–1.05**	**1.03**	**1.02–1.04**

Marital status	Currently married	REF		REF	
	Never married	0.79	0.66–0.94	**0.80**	**0.65–0.99**
	Separated, divorced, or widowed	1.15	0.84–1.58	1.39	0.93–2.06

Education	Primary school or less	REF			
	Elementary or high school completed	0.95	0.80–1.14		
	College degree or higher education	1.04	0.85–1.26		

Daily servings of red meat and chicken	0-1			REF	
	1-2			0.95	0.79–1.14
	2-3			**0.69**	**0.54–0.88**
	3+			0.95	0.76–1.19

Daily hours spent watching TV	0-1			REF	
	1–3			1.03	0.75–1.42
	3–5			1.25	0.90–1.74
	5+			1.20	0.85–1.69

Levels of physical activity	None			REF	
	Low			0.99	0.81–1.21
	Moderate			**0.77**	**0.60–0.98**
	High			0.87	0.70–1.06

Obesity	Not obese			REF	
	Obese			**1.55**	**1.30–1.83**

History of diagnosis with diabetes	No			REF	
	Yes			**1.16**	**0.83–1.63**

Diagnosis of chronic condition	No			REF	
	Yes			0.89	0.64–1.24

*AOR for age should be considered as for an increase of one year.

Odds ratios were adjusted for sociodemographic characteristics: sex, age, marital status, and education.

AOR: adjusted odds ratios; CI: confidence intervals; REF: reference.

**Table 5:** Multivariate logistic regression for sociodemographic characteristics, risk factors, and undiagnosed hypertension, in Saudi Arabia, of males and females aged 15 years or older, 2013.

Sociodemographic and risk factors	Categories	Sociodemographic model		Full adjusted model	
		AOR	95% CI	AOR	95% CI
Sex	Males	REF		REF	
	Females	**0.54**	**0.44–0.66**	**0.54**	**0.44–0.67**

Age∗	Age	**1.06**	**1.05-1.06**	**1.05**	**1.05-1.06**

Marital status	Currently married	REF			
	Never married	0.77	0.57–1.03	0.78	0.58–1.05
	Separated, divorced, or widowed	1.17	0.85–1.61	1.13	0.81–1.57

Education	Primary school or less	REF			
	Elementary or high school completed	0.96	0.75–1.23	0.97	0.76–1.25
	College degree or higher education	0.77	0.59–1.02	0.79	0.60–1.03

History of diagnosis with prediabetes	No			REF	
	Yes			0.73	0.41–1.31

History of diagnosis with diabetes	No			REF	
	Yes			**1.46**	**1.09–1.94**

*AOR for age should be considered as for an increase of one year.

Odds ratios were adjusted for sociodemographic characteristics: sex, age, marital status, and education.

AOR: adjusted odds ratios; CI: confidence intervals; REF: reference.

## References

[1] Wang H, Dwyer-Lindgren L, Lofgren KT (2012). Age-specific and sex-specific mortality in 187 countries, 1970–2010: a systematic analysis for the Global Burden of Disease study 2010. *The Lancet*.

[2] Messerli FH, Williams B, Ritz E (2007). Essential hypertension. *The Lancet*.

[3] Singh TK, Arya V, Navaratnarajah N (2014). Chronic kidney disease and cardiovascular disease: a focus on primary care. *Cardiovascular & Hematological Disorders-Drug Targets*.

[4] He J, Whelton PK (1999). Elevated systolic blood pressure as a risk factor for cardiovascular and renal disease. *Journal of Hypertension*.

[5] Elliott WJ (2003). The economic impact of hypertension. *The Journal of Clinical Hypertension*.

[6] Kearney PM, Whelton M, Reynolds K, Muntner P, Whelton PK, He J (2005). Global burden of hypertension: analysis of worldwide data. *The Lancet*.

[7] Institute for Health Metrics and Evaluation (IHME) GBD arrow Diagram, Saudi Arabia. Risk of deaths.1990–2010. http://www.healthmetricsandevaluation.org/gbd/visualizations/gbd-arrow-diagram.

[8] Institute for Health Metrics and Evaluation (IHME) (2013). *Stacked bar chart, Saudi Arabia. Deaths.1990–2010 [Internet]*.

[9] Al-Nozha MM, Abdullah M, Arafah MR (2007). Hypertension in Saudi Arabia. *Saudi Medical Journal*.

[10] Al-Hamdan N, Saeed A, Kutbi A, Choudhry AJ, Nooh R (2010). Characteristics, risk factors, and treatment practices of known adult hypertensive patients in Saudi Arabia. *International Journal of Hypertension*.

[11] Saeed AA, Al-Hamdan NA, Bahnassy AA, Abdalla AM, Abbas MAF, Abuzaid LZ (2011). Prevalence, awareness, treatment, and control of hypertension among Saudi adult population: a national survey. *International Journal of Hypertension*.

[12] Craig CL, Marshall AL, Sjöström M (2003). International physical activity questionnaire: 12-country reliability and validity. *Medicine and Science in Sports and Exercise*.

[13] National Heatlth and Nutrition Examination Survey (NHANES) (2009). *Health Tech/ Blodd Pressure Procedures Manual. [Internet]*.

[14] Causland FRM, Sacks FM, Forman JP (2014). Marital status, dipping and nocturnal blood pressure: results from the dietary approaches to stop hypertension trial. *Journal of Hypertension*.

[15] Lipowicz A, Lopuszanska M (2005). Marital differences in blood pressure and the risk of hypertension among Polish men. *European Journal of Epidemiology*.

[16] Alwan H, Pruijm M, Ponte B (2014). Epidemiology of masked and white-coat hypertension: the family-based SKIPOGH study. *PloS ONE*.

[17] (1977). Race, education and prevalence of hypertension. *American Journal of Epidemiology*.

[18] Fuchs FD, Moreira LB, Moraes RS, Bredemeier M, Cardozo SC (1994). Prevalence of systemic arterial hypertension and associated risk factors in the Porto Alegre metropolitan area. Populational-based study. *Arquivos Brasileiros de Cardiologia*.

[19] Ministry of Health (2013). *Kingdom of Saudi Arabia Projected Population*.

[20] Institute for Health Metrics and Evaluation (IHME) (2013). *Healthy Years Lost vs Life Expectancy, Saudi Arabia. Risk of Deaths. 1990–2010*.

